# Supplementary material for: Thermoregulation Effects of Phoneutria nigriventer Isolated Toxins in Rats
Source: Toxins (Basel). 2024 Sep 18;16(9):398. doi: 10.3390/toxins16090398 (PMC11435823; doi:10.3390/toxins16090398)
Supplement: Supplementary file 1 [file toxins-16-00398-s001.zip › toxins-3103699-supplementary.pdf]

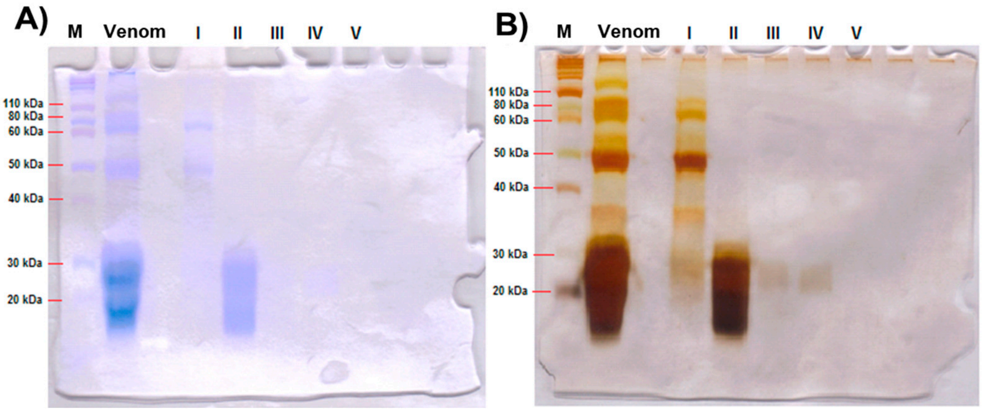

Figure S1. SDS-PAGE electrophoresis (Comassie Blue and silver nitrate).

SDS PAGE (15% polyacrylamide gel electrophoresis). (a) *Phoneutria nigriventer* total venom and its five pools stained with Coomassie, (b) *Phoneutria nigriventer* crude venom and its five pools stained with silver. M - molecular weight standard, I - Pool I, II - Pool II, III - Pool III, IV - Pool IV and V - Pool V. For analysis, both the total venom and the fractions from gel filtration were subjected to 15% polyacrylamide gel electrophoresis in the presence of SDS (Laemmli, 1970). The samples were diluted in the same volume of 5x sample buffer (350 mM Tris / HCl, 10% SDS, 30% glycerol, 1.2 mg bromophenol blue, 9.3% 2-mercaptoethanol) and (AccuBlock ™ Digital Dry Bath, Labnet, Labnet Internat International, Inc.) for 5 minutes. The samples were applied to the gel and subjected to a constant electric current of 110 volts. The gels were stained with Coomassie blue R-250 and also with silver.

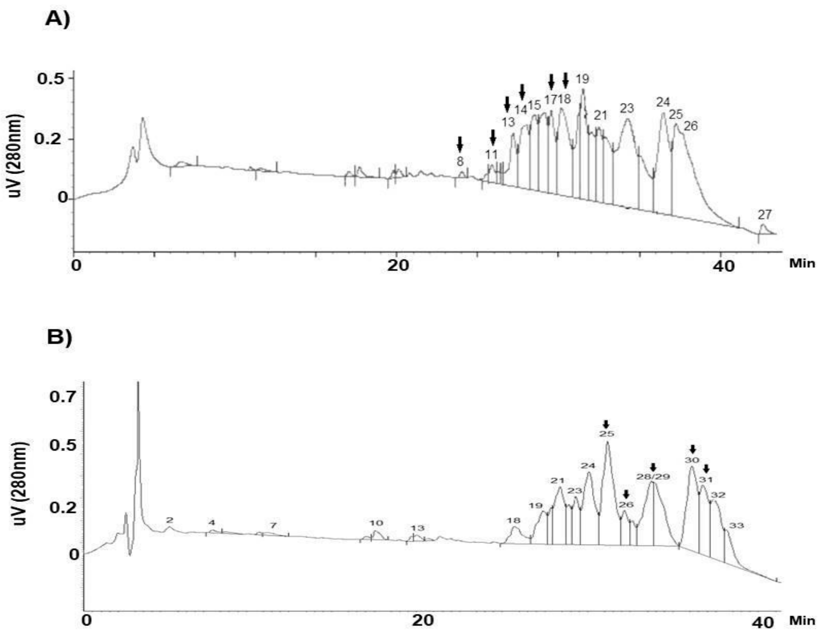

Figure S2. Purification of the peaks (II and III) of *Phoneutria nigriventer* by high-performance liquid chromatography.

Chromatographic profile of peaks II (A) and III (B) of *Phoneutria nigriventer* venom. Reverse phase chromatography (C18 column) was used for fractionation, with a gradient of 5% to 100% solution B over 45

minutes, with a flow rate of 1 mL/min and a wavelength of 280 nm. The arrows indicate the peaks that were tested in the subsequent experiments. Peak II showed 32 peaks, while peak III showed 33 peaks. In order to identify the compounds that showed activity, pool III peaks were repurified by high performance liquid chromatography (CLAE - Shimadzu 10-A, ClassVP software) using a reverse phase C18 column (LiChroCart 250-4 μm 250 mm X 46 mm) , monitored by a variable UV detector set at 214 nm. The following solutions were used for elution: (A) 0.1% TFA (trifluoroacetic acid) (MERCK) in ultrapure water; (B) 10% of mobile phase A in acetonitrile.

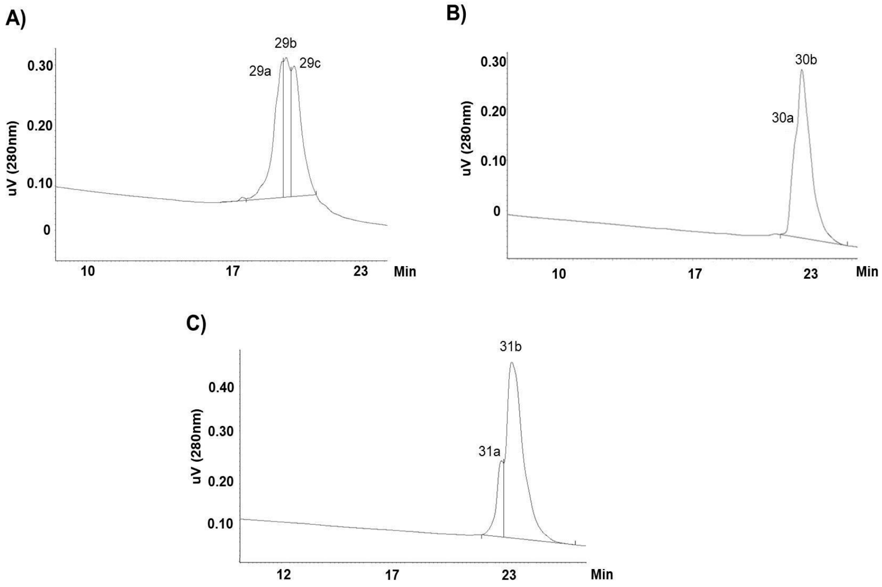

Figure S3. Repurification of the HPLC active peaks (28/29, 30 and 31) from pool III of *Phoneutria nigriventer* for evaluation on the body temperature.

Considering that the peaks tested from pool III showed thermal activity in rats (peaks 28/29, with hypothermic activity, and peaks 30 and 31, with hyperthermic activity), these materials were purified by reverse phase chromatography. Chromatographic profile of peaks 28/29 (A), 30 (B), and 31(C) of pool III of the *Phoneutria nigriventer* venom by HPLC. Purification of peaks with hypothermic activity, using HPLC coupled with C18 column reverse phase, with a gradient of 25% to 100% of solution B in 40 minutes, with a flow of 1 mL/min, monitored at a length of 214 nm.

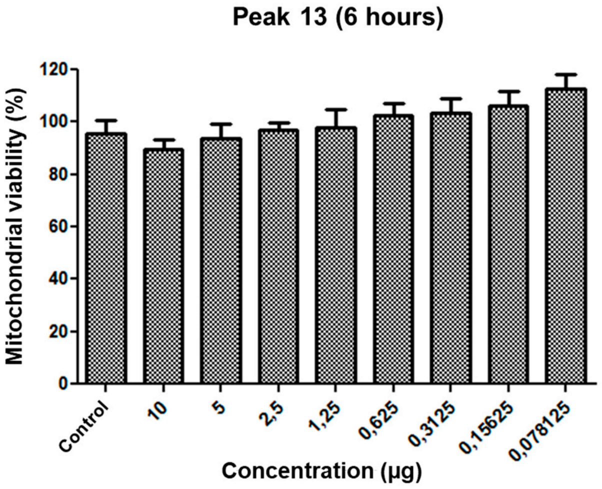

Figure S4 Analysis of cell viability in culture of human glioblastoma U87 from *Phoneutria nigriventer* venom peak 13 with action on thermoregulation in rats (6-hour).

Graph representing the percentage of cells (human glioblastoma U87) alive through the MTT colorimetric test and by exclusion using the trypan blue method after an incubation period of 24 hours in different concentrations in triplicate of peak 13 of pool II of the venom of *Phoneutria nigriventer*.

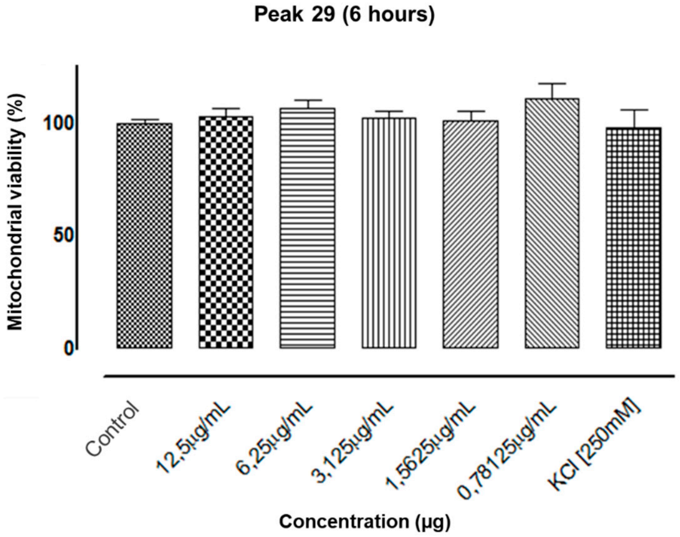

Figure S5 Analysis of cell viability in culture of human glioblastoma U87 from *Phoneutria nigriventer* venom peak 29 with action on thermoregulation in rats (6-hour).

Neuro-basal cell viability with incubation periods of 6 hours with peak 29. Percentage of cells (neuro-basal) alive after an incubation period of 6 hours at different concentrations (12.5 µg/mL; 6.25 µg /mL; 3.125 µg/mL; 1.5625 µg/mL and 0.78125 µg/mL) in triplicate of peak 29 of pool III of *Phoneutria nigriventer* venom.

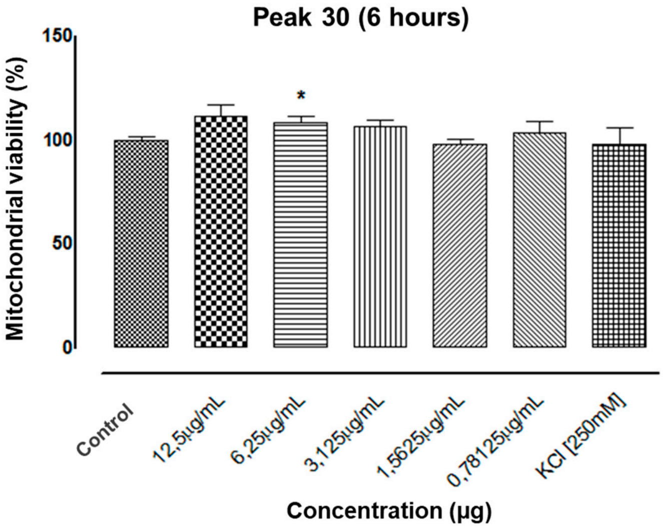

Figure S6 Analysis of cell viability in culture of human glioblastoma U87 from *Phoneutria nigriventer* venom peak 30 with action on thermoregulation in rats (6-hour).

Neuro-basal cell viability with incubation periods of 6 hours with peak 30. Percentage of cells (neuro-basal) alive after an incubation period of 6 hours at different concentrations (12.5 µg/mL; 6.25 µg /mL; 3.125 µg/mL; 1.5625 µg/mL and 0.78125 µg/mL) in triplicate of peak 30 of pool III of *Phoneutria nigriventer* venom.

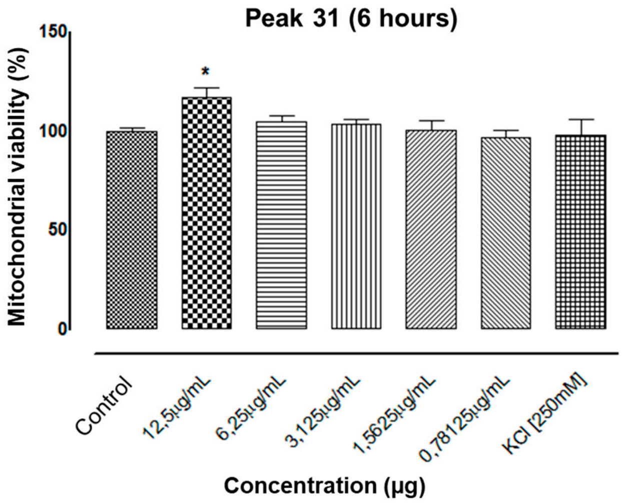

Figure S7 Analysis of cell viability in culture of human glioblastoma U87 from *Phoneutria nigriventer* venom peak 31 with action on thermoregulation in rats (6-hour).

Neuro-basal cell viability with incubation periods of 6 hours with peak 31. Percentage of cells (neuro-basal) alive after an incubation period of 6 hours at different concentrations (12.5 µg/mL; 6.25 µg /mL; 3.125 µg/mL; 1.5625 µg/mL and 0.78125 µg/mL) in triplicate of peak 31 of pool III of *Phoneutria nigriventer* venom.

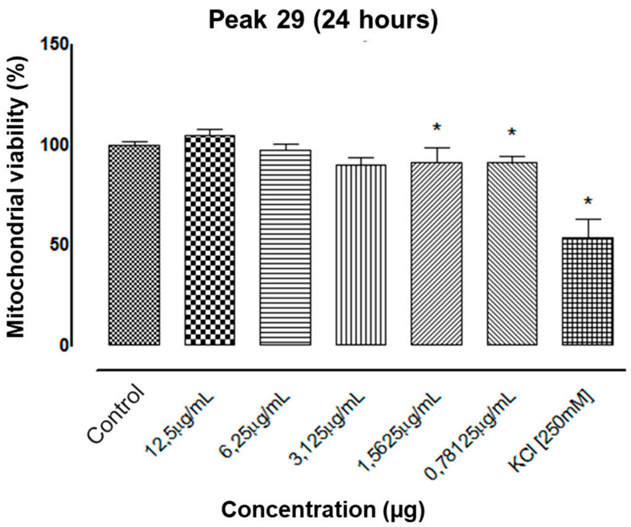

Figure S8 Analysis of cell viability in culture of human glioblastoma U87 from *Phoneutria nigriventer* venom peak 29 with action on thermoregulation in rats (24-hour).

Neuro-basal cell viability with 24-hour incubation periods with peak 29. Percentage of cells (neuro-basal) alive after 24-hour incubation period at different concentrations (12.5 µg/mL; 6.25 µg /mL; 3.125 µg/mL; 1.5625 µg/mL and 0.78125 µg/mL) in triplicate of peak 29 of pool III of *Phoneutria nigriventer* venom.

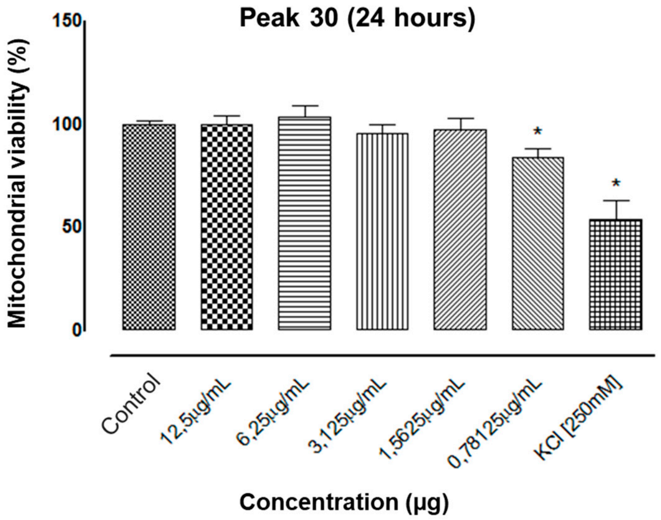

Figure S9 Analysis of cell viability in culture of human glioblastoma U87 from *Phoneutria nigriventer* venom peak 30 with action on thermoregulation in rats (24-hour).

Neuro-basal cell viability with 24-hour incubation periods with peak 30. Percentage of cells (neuro-basal) alive after 24-hour incubation period at different concentrations (12.5 µg/mL; 6.25 µg /mL; 3.125 µg/mL; 1.5625 µg/mL and 0.78125 µg/mL) in triplicate of peak 30 of pool III of *Phoneutria nigriventer* venom.

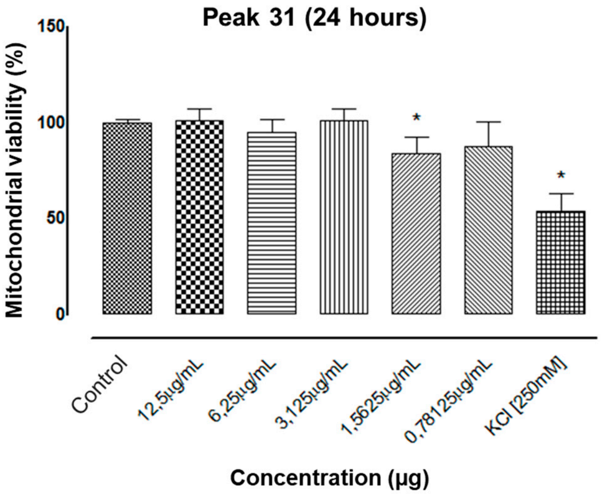

Figure S10 Analysis of cell viability in culture of human glioblastoma U87 from *Phoneutria nigriventer* venom peak 31 with action on thermoregulation in rats (24-hour).

Neuro-basal cell viability with 24-hour incubation periods with peak 31. Percentage of cells (neuro-basal) alive after 24-hour incubation period at different concentrations (12.5 µg/mL; 6.25 µg /mL; 3.125 µg/mL; 1.5625 µg/mL and 0.78125 µg/mL) in triplicate of peak 31 of pool III of *Phoneutria nigriventer* venom.
